# 

**Published:** 1899-01-14

**Authors:** 


NO DRUGS OR ALKALIES USED.

GLASGOW WESTERN INFIRMARY

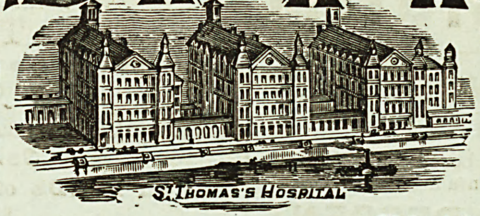

ST THOMAS'S HOSPITAL

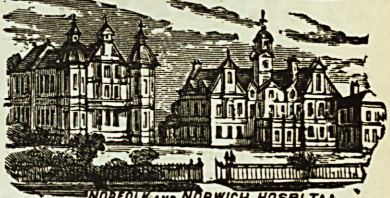

NORFOLK AND NORWICH HOSPITAL

No. 642. Vol. XXV. [Registered as a Newspaper.] SATURDAY, JAN. 14TH, 1899. [Subscription Terms, see p. 258.] PRICE TWOPENCE.

